# Inhibition of Galectin-3 Alleviates Cigarette Smoke Extract-Induced Autophagy and Dysfunction in Endothelial Progenitor Cells

**DOI:** 10.1155/2019/7252943

**Published:** 2019-10-13

**Authors:** ChongZhe Pei, Xiaoyan Wang, Yanjun Lin, Lu Fang, Shu Meng

**Affiliations:** ^1^Department of Cardiology, Xinhua Hospital, School of Medicine, Shanghai Jiaotong University, China; ^2^Department of Cardiology, Zhongshan Hospital, School of Medicine, Fudan University, China; ^3^Haematopoiesis and Leukocyte Biology Laboratory, Baker Heart and Diabetes Research Institute, Melbourne, VIC, Australia

## Abstract

Endothelial progenitor cells (EPCs) have the potential to repair damaged blood vessels and promote angiogenesis. Smoking, an important risk factor for cardiovascular diseases, is associated with impaired functions of EPCs. However, the underlying mechanisms remain unclear. The aim of the study was to investigate the effects of cigarette smoke extract (CSE) on autophagy and dysfunction of EPCs and the involvement of galectin-3 in its effects. EPCs were treated with 8% CSE for 24 h (without affecting cell viability). EPC functions were assessed by tube formation and migration capacity and intracellular ROS and eNOS expression. Autophagy was assessed by autophagic protein expression by Western blotting and immunofluorescence microscopy and autophagosome accumulation by transmission electron microscopy. Galectin-3 expression was measured by real-time PCR, Western blotting, and immunofluorescence microscopy, while phospho-AMPK and phospho-mTOR were measured by Western blotting. EPCs were transfected by shRNA-Gal-3 or shRNA-NC before treatment with CSE to examine the effects of galectin-3 on CSE-induced autophagy and dysfunction of EPCs. CSE-treated EPCs showed decreased tube formation and migration ability and eNOS expression but increased oxidative stress. CSE also induced autophagy which was characterized by a decrease in p62 protein, an increase in LC3B-II/I ratio, and accumulation of autophagosomes. CSE upregulated galectin-3 expression on EPCs. Inhibition of galectin-3 abrogated CSE-induced autophagy and dysfunction of EPCs. CSE activated phospho-AMPK and inhibited phospho-mTOR, and inhibition of galectin-3 abolished CSE's effect on activating phospho-AMPK and inhibiting phospho-mTOR. In conclusion, our results suggest that galectin-3 mediates CSE-induced EPC autophagy and dysfunction, likely via the AMPK/mTOR signaling pathway.

## 1. Introduction

Smoking is an important risk factor for many cardiovascular diseases [1–4]. Smoking accelerates cardiovascular events through causing endothelial dysfunction, arterial stiffness, inflammation, and lipid modification [5, 6], and endothelial dysfunction is one of the earliest pathological effects of cigarette smoking [7, 8]. Accumulating studies documented that smoking had detrimental effects on endothelial progenitor cells (EPCs), which are bone marrow-derived stem cells and have the potential to differentiate into endothelial cells to repair damaged blood vessels after myocardial and cerebral infarction [9–12]. Studies showed that the number of EPCs was reduced and EPC functions were impaired in smokers compared with nonsmokers and reduced EPC levels were restored following smoking cessation [13–16]. Active smoking-associated EPC alterations could contribute to impaired cardiac function recovery after reperfusion therapy in smokers [17].

Autophagy refers to the degradation of intracellular structures, including macromolecules such as organelles, proteins, and nucleic acids, by intracellular lysosomes, providing raw materials for cell reconstruction, regeneration, and repair, thereby ensuring the metabolic balance of cells [18]. Altered autophagy has been implicated in diseases such as cancer, neurodegenerative disorders, and cardiovascular diseases [19]. Regulating autophagy has been applied in many aspects, such as preventing apoptosis [20], enhancing antitumor activity [21], improving cell survival [22], and promoting cell proliferation [23]. Studies showed that cigarette smoke extract (CSE) induced the level of autophagy in retinal pigment epithelial cells [24], and in human bronchial epithelium [25]. However, whether autophagy is dysregulated by CSE in EPCs is unknown.

Galectin-3 is one of the important members of the galectin family. Galectin-3 is involved in multiple pathophysiological processes such as cell growth, adhesion, proliferation, apoptosis, angiogenesis, inflammation, fibrosis, and metastasis [26–28] and the pathogenesis of many diseases [29, 30]. Galectin-3 is widely distributed in epithelial cells, endothelial cells, fibroblasts, and macrophages, and its expression was higher in EPCs than in endothelia cells [31]. Recent studies showed that galectin-3 played an important role in mediating autophagy in protecting cells against endomembrane damage associated with lysosomal dysfunction [32, 33]. So, we hypothesized that galectin-3 regulated autophagy in EPCs.

Thus, the aims of the present study were to examine whether galectin-3 mediates the effects of CSE on EPC function and autophagy and the underlying signaling pathways.

## 2. Materials and Methods

### 2.1. Cell Culture

The use of human blood conformed to the principles outlined in the Declaration of Helsinki, and written informed consent was obtained from each donor. EPCs were derived from peripheral blood mononuclear cells (PBMCs) of healthy donors. PBMCs were isolated by density gradient centrifugation with Ficoll-Isopaque Plus (Histopaq-1077, density 1.077 g/mL, Sigma, USA), and cells were plated onto culture dishes in endothelial growth medium (EBM-2-MV BulletKit, Lonza, Switzerland), with supplements (hydrocortisone, R3-insulin-like growth factor 1, human endothelial growth factor, vascular endothelial growth factor, human fibroblast growth factor, GA-100, ascorbic acid, heparin, and 20% fetal bovine serum) at 37°C in a 5% CO2 incubator. After 4 days, nonadherent cells were removed by washing with phosphate-buffered saline (PBS). The medium was changed every three days over a three-week period. After 14 days of culture, the cells were incubated with fluorescein isothiocyanate-conjugated lectin from Ulex europaeus agglutinin 1 (FITC-UEA-1) (Sigma, Germany), and 1,19-dioctadecyl-3,3,3939-tetramethylindocar-bocyanine perchlorate-labeled acetylated low-density lipoprotein (LDL-ac-Dil) (Sigma, Germany). Cells positive for both LDL-ac-Dil and UEA-1 were identified as EPCs. The purity of the EPCs was analyzed by flow cytometry after staining with CD34, CD133, and KDR (all from BioLegend Inc., USA).

### 2.2. Preparation of Cigarette Smoke Extract

Cigarette smoke extract (CSE) was prepared as previously described [34]. Briefly, 400 mL of cigarette smoke (containing 12 mg of tar and 2.5 mg of nicotine per cigarette; Da Qianmen, Shanghai, China) was drawn into a 50 mL plastic syringe through a 3-way stopcock and mixed with 20 mL of EBM-2-MV by vigorous shaking. The cigarette filter was removed during the procedure to prepare the CSE. One cigarette was used per 20 mL CSE, and CSE was prepared not earlier than 30 min before being used in experiments. The CSE solution was filtered through an aseptic 0.22 *μ*m filter and considered as a 100% extract.

### 2.3. Cell Viability Assay

EPC viability was assessed by using a cell counting kit (CCK-8; Dojindo, Kumamoto, Japan). Test cells (5 × 10^3^) were plated into 96-well plates. For choosing an optimal concentration and an incubation time, EPCs were treated for 24 h with several concentrations of CSE (0%, 1%, 2%, 5%, 8%, 10%, and 15%) and with one selected concentration for different time periods (6, 12, 24, and 48 h). The medium was removed, and 100 *μ*L of fresh medium containing 10 *μ*L water-soluble tetrazolium (WST-8) reagent was added to each well. The cells were incubated with WST-8 reagent at 37°C for 2 h. The cell viability in each well was determined by measuring the absorbance at 450 nm using a microplate reader. Data were expressed as the optical density value. Each experiment was performed 4 times independently.

### 2.4. Galectin-3 Short Hairpin RNA (shRNA) Construction and Infection

shRNA-galectin-3 (shRNA-Gal-3) and relative negative control (NC) were designed and synthesized by Shanghai GenePharma Co. Ltd., Shanghai, China. For shRNA-galectin-3 construction, one RNA interference vector pGLV3/H1/GFP&Puro and three specific shRNAs for galectin-3 (shRNA-Gal-3-599, shRNA-Gal-3-651, and shRNA-Gal-3-683) were designed and synthesized based on the human galectin-3 target sequence (NCBI gene ID: 3958). A negative control was produced according to the same design principle for shRNA. The above three shRNAs and the shRNA-NC were transfected into EPCs using X-tremeGENE HP DNA transfection reagent (Roche, Basel, Switzerland) following the manufacturer's protocol. After 24 h, Western blot was used to find out the optimal shRNA and shRNA-Gal-3-651 (shRNA-1): 5′-GCCACTGATTGTGCCTTATAA-3′ was selected.

### 2.5. Tube Formation Assay

Serum-starved EPCs were seeded onto Matrigel-coated plates (BD Biosciences, USA) in EBM medium and incubated at 37°C for 24 h, and tubular structures of EPCs in the Matrigel were analyzed by phase-contrast microscopy. To quantify the length of newly formed tubes, 6 random phase-contrast photomicrographs per well were taken and the length of each tube was measured using Quantity One Image software.

### 2.6. Migration Assay

Different groups of EPCs (1 × 10^5^/mL in 200 *μ*L) were resuspended in serum-free EBM medium, and cells were loaded into the upper transwell chambers (Corning, Corning Incorporated Life Science, USA), and the lower chambers were filled with 750 *μ*L medium (EBM-2-MV BulletKit, Lonza). The assays were conducted over a 24 h incubation period at 37°C in an incubator equilibrated with 5% CO2. The membrane was then washed gently with PBS, and nonmigrated cells were removed with cotton balls from the upper side of the membrane while migrated cells were fixed with 4% paraformaldehyde. The membrane was then stained by using 0.1% crystal violet solution for 30 min. Migrated EPCs were counted under a microscope (CX31, Olympus Japan) in 4 random high-power fields (×100) in each membrane coverage. All groups were performed in triplicate.

### 2.7. Detection of Intracellular Reactive Oxygen Species (ROS)

EPCs were seeded onto cover slips and then treated with 8% CSE for 24 h to measure intracellular ROS. The production of ROS in EPCs was determined by immunofluorescence microscopy, using DHE staining (10 *μ*mol/L, Beyotime, China). The nuclei were stained with 4′,6-diamino-2-phenylindole, dihydro-chloride (DAPI) (100 mg/L, eBioscience, USA). The cells were incubated with DHE for 30 min and DAPI for 15 min at room temperature. Fluorescent images were captured by inverted fluorescence microscope (TE2000U, Nikon, Japan). Mean fluorescence intensity was calculated by averaging area intensities of cells. The result was expressed as integrated optical density (IOD)/area by using Image-Pro Plus 6.0 software according to the standard protocol.

### 2.8. Protein Extraction and Western Blot

Total cellular protein was extracted in RIPA lysis buffer (Beyotime, Shanghai, China) supplemented with 1% phenylmethanesulfonyl fluoride (PMSF) (Beyotime, Shanghai, China) and 1% phosphatase inhibitor (Beyotime, Shanghai, China). Equal amounts of protein (30 *μ*g) were separated through a 10% or 12% SDS-PAGE and transferred to a PVDF membrane. Membranes were first probed with primary antibodies for p65, LC3B, galectin-3, adenosine monophosphate kinase (AMPK), phosphate-AMPK (Thr172), mammalian target of rapamycin (mTOR), phosphate-mTOR (Ser2448), and GAPDH (all the above 8 antibodies: 1 : 1000; from Cell Signaling Technology, USA) and then incubated with anti-rabbit or anti-mouse secondary antibodies (1 : 1000; Beyotime, Shanghai, China). All signals were detected by the Molecular Imager ChemiDoc™ XRS+ System (Bio-Rad, Hercules, CA, USA). p65, LC3B, and galectin-3 were normalized by GAPDH levels. Phospho-AMPK and phosphate-mTOR were normalized by total AMPK and total mTOR.

### 2.9. RNA Isolation and Real-Time PCR

Total RNA was isolated from cells using TRIzol (Invitrogen, USA). Real-time PCR was performed to determine gene expression of galectin-3. The primer sequences are shown in Table 1. The PCR reaction was performed as follows: stage 1, 94°C for 2 min; stage 2, 94°C for 20 s; and 60°C for 34 s. Stage 2 was repeated for 40 cycles. Real-time PCR was performed using the SYBR and ROX PCR master mix (Takara, Japan) with the Applied Biosystems ABI7500 Real-time PCR System (Applied Biosystems, USA). GAPDH was used as an endogenous control. All samples were normalized to internal controls, and the relative expression level was calculated using the 2^−ΔΔCt^ analysis method.

### 2.10. Immunofluorescence

For immunofluorescence, different groups of EPCs were blocked with 1% bovine serum albumin (BSA) (Sigma, USA)/PBS for 1 h at room temperature and cells were then incubated for 1 h at room temperature with anti-human eNOS (unconjugated) and a rabbit anti-human LC3B antibody (unconjugated) and anti-human galectin-3 (Conjugate with Alexa Fluor® 647) (1 : 100; all from Abcam, USA). After being washed with PBS containing 0.1% Tween-20, samples for eNOS and LC3B were incubated with a secondary antibody (Alexa Fluor 488 mouse-anti-rabbit IgG at 1 : 200, Invitrogen, USA) for 2 h at room temperature. Following fixation, the cell nucleus was stained with 4,6-diamino-2-phenylindole (DAPI) (eBioscience, USA). Fluorescent images were captured by an inverted fluorescence microscope (TE2000U, Nikon, Japan). Mean fluorescence intensity was calculated by averaging area intensities of cells. The result was expressed as integrated optical density (IOD)/area by using Image-Pro Plus 6.0 software according to the standard protocol.

### 2.11. Transmission Electron Microscopy (TEM)

For TEM, EPCs were fixed with 2.5% glutaraldehyde in phosphate buffer and stored at 4°C overnight. The EPCs were further fixed with 1% osmium tetroxide and stained with 1% uranyl acetate, followed by a gradient dehydration step using ethanol and acetone. The EPCs were then embedded in araldite. Ultrathin sections were obtained (50 nm) and placed on uncoated copper grids. Images were examined with an H-7500 transmission electron microscope (Hitachi, Tokyo, Japan). Autophagosomes from three sections per cell and three cells per group were counted.

### 2.12. Statistical Analysis

Variables were reported as mean ± SD. Each cell experiment was performed at least three times. Data were compared using 2-tailed Student's *t*-test for two independent samples or one-way ANOVA followed by Kruskal-Wallis *H* test for more than two groups. *p* < 0.05 was considered statistically significant. All statistical analyses were performed with IBM SPSS Statistics for Windows 24.0 (SPSS Inc., Chicago, IL, USA).

## 3. Results

### 3.1. Characterization of Cultured Human EPCs

**Figure 1:**
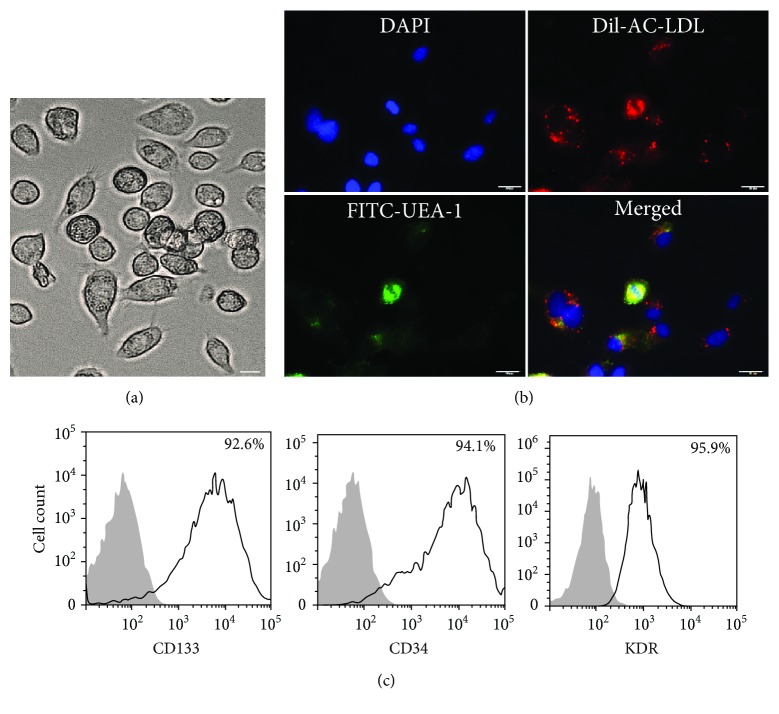
Characterization of cultured human EPCs. PBMC-derived EPCs with a spindle shape formed clones (a) (200x). EPCs were shown to simultaneously endocytose DiI-ac-LDL (red) and bind fluorescein isothiocyanate UEA-1 (lectin, green), and a merged photo was also presented (b) (200x). FACS analysis showed high expression of CD133, CD34, and KDR on EPCs (c). Scale bar = 50 *μ*m.

Ten days after plating, adherent EPCs with a spindle shape formed clones (Figure 1(a)). Most EPCs were shown to simultaneously endocytose DiI-ac-LDL (red) and bind to fluorescein isothiocyanate UEA-1 (lectin, green) (Figure 1(b)). FACS analysis showed high expression of CD133, CD34, and KDR on the surface of EPCs after 14 days of culture (Figure 1(c)).

### 3.2. The Effects of CSE on Cell Viability

**Figure 2:**
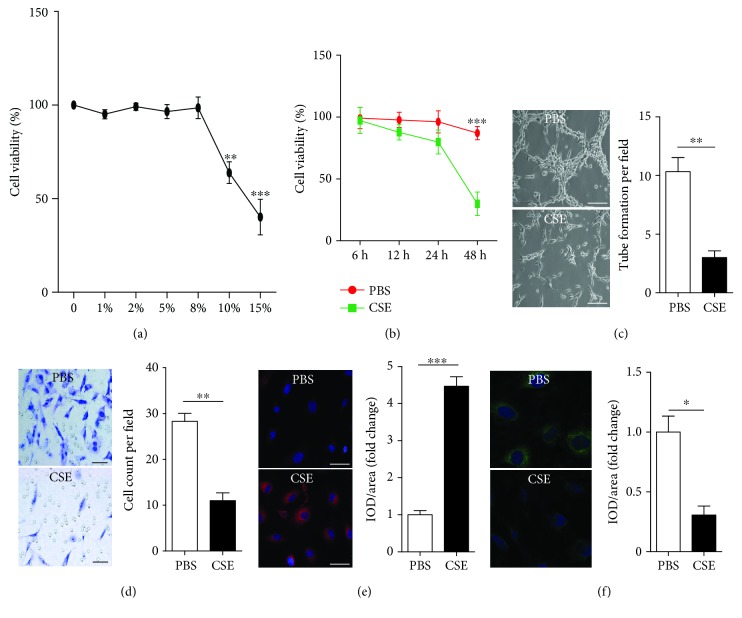
CSE induced EPC dysfunction. EPCs were first incubated with different concentrations (0%, 1%, 2%, 5%, 8%, 10%, and 15%) of CSE for 24 h to choose an optimal concentration and then incubated with 8% CSE over an extended period to choose an optimal experimental time. Cell viability was assessed with CCK-8 (a, b). EPCs were treated with PBS, CSE (8%) for 24 h. EPC tube formation capacity was analyzed with a tube formation assay (C). EPC migration capacity was analyzed with a migration assay (d). Intracellular ROS was examined by DHE staining (e). Intracellular eNOS was examined by immunofluorescence (f). Data are represented as mean ± SD. *n* ≥ 3. ^∗∗^*p* < 0.01 and ^∗∗∗^*p* < 0.001 vs. the PBS group. Scale bar = 100 *μ*m.

Since we focused on the effect of CSE on autophagy in EPCs in the present study, we first assessed the effect of different concentrations and different incubation times of CSE on cell viability of EPCs in order to choose an optimal concentration and an optimal incubation time without affecting cell viability of EPCs. EPCs were treated with 1%, 2%, 5%, 8%, 10%, and 15% of CSE for 24 h. As shown in Figure 2(a), 1-8% CSE for 24 h had little influence on cell viability, while 10% and 15% of CSE significantly decreased cell viability of EPCs. Then we used 8% of CSE for different time periods (6, 12 h, 24 h, and 48 h). As shown in Figure 2(b), 8% CSE had little influence on cell viability within 24 h; however, cell viability of EPCs was reduced significantly after treatment with 8% of CSE for 48 h. Thus, for the subsequent experiments, 8% CSE and an incubation time of 24 h were used.

### 3.3. CSE Induced EPC Dysfunction

Treatment with 8% CSE for 24 h decreased EPC tube formation capacity (Figure 2(c)) and migration capacity (Figure 2(d)) and increased intracellular ROS production (Figure 2(e)) and decreased intracellular eNOS expression (Figure 2(f)) compared with PBS control.

### 3.4. CSE Enhanced Autophagy in EPCs

**Figure 3:**
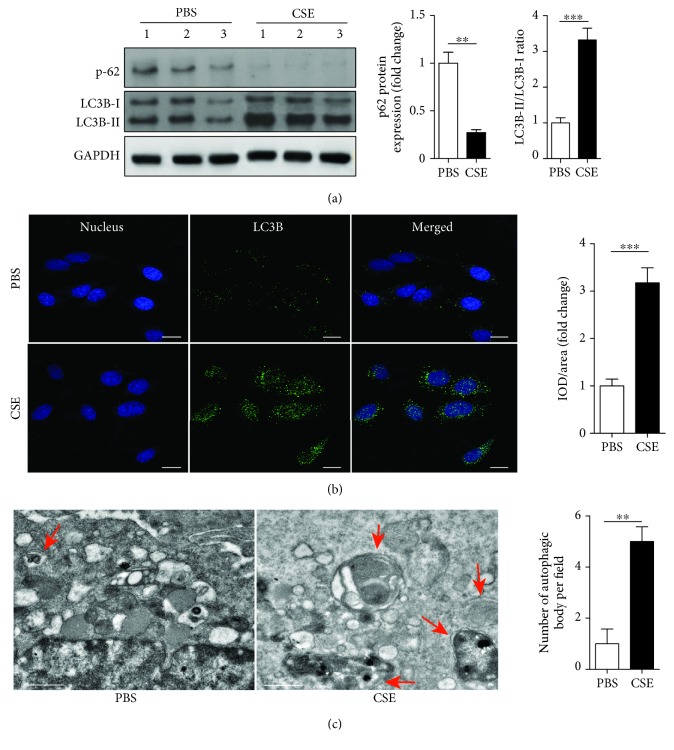
CSE induced EPC autophagy. EPCs were treated with PBS, CSE (8%) for 24 h. Western blots of p-62, LC3B-I, and LC3B-II and quantification of P-62 and LCB-II/I (a). Immunofluorescence and quantification of LC3B expression on the cell membrane of EPCs (b) (scale bar = 100 *μ*m). TEM and quantification of autophagosomes in EPCs (c) (scale bar = 1 *μ*m). Data are represented as mean ± SD. *n* ≥ 3. ^∗∗^*p* < 0.01 and ^∗∗∗^*p* < 0.001 vs. the PBS group.

Autophagic activity was evaluated by measuring autophagic protein expression using Western blot and immunofluorescence and counting the number of autophagosomes in EPCs using TEM. Expression of p62 protein was significantly downregulated, while the LC3B-II/I ratio was significantly upregulated in EPCs from the CSE group compared with PBS control (Figure 3(a)). The IOD/area of LC3B was significantly upregulated in the CSE group compared with PBS control (Figure 3(b)). TEM demonstrated that the mean number of autophagosomes per cell was significantly increased in the CSE group compared with PBS control (Figure 3(c)).

### 3.5. CSE Upregulated Galectin-3 Expression in EPCs

**Figure 4:**
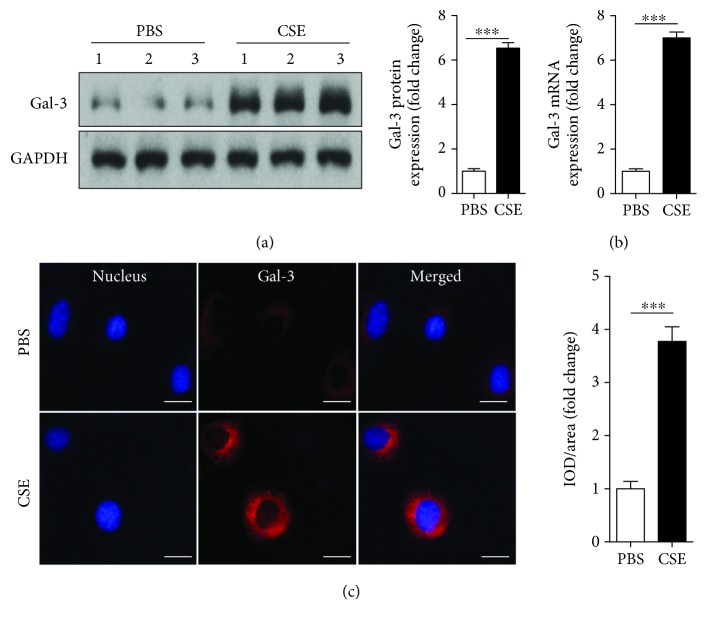
CSE upregulated galectin-3 expression in EPCs. PBMC-derived EPCs were treated with PBS, CSE (8%) for 24 h. Western blots and quantification of galectin-3 (a). Relative gene expression of galectin-3 in EPCs was determined by real-time PCR (b). Immunofluorescence was used to confirm galectin-3 expression on the cell membrane of EPCs (c). Data are represented as mean ± SD. *n* ≥ 3. ^∗∗∗^*p* < 0.001 vs. the PBS group. Scale bar = 100 *μ*m.

Next, we investigated the effect of CSE on galectin-3 expression on EPCs. EPCs were treated with CSE (8%) for 24 h. Galectin-3 protein and mRNA were upregulated by CSE incubation (Figures 4(a) and 4(b)). Immunofluorescence also confirmed that CSE (8%) increased galectin-3 expression on the surface of EPCs (Figure 4(c)).

### 3.6. Inhibition of Galectin-3 Attenuated CSE-Induced EPC Autophagy

**Figure 5:**
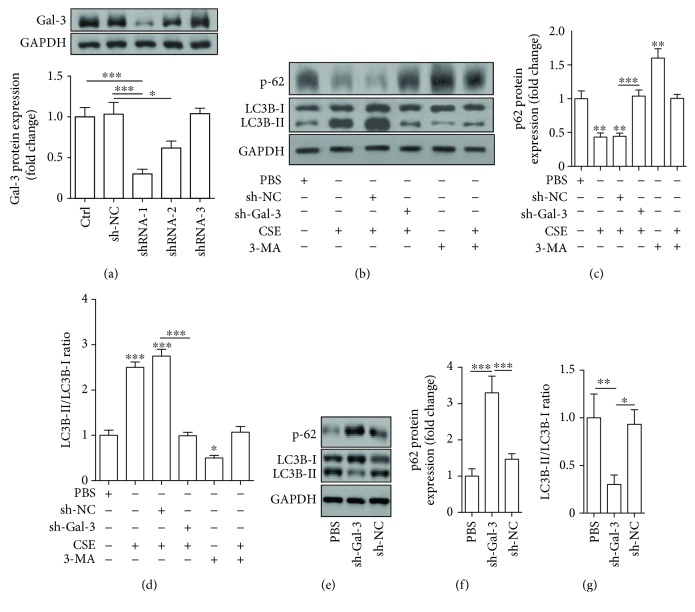
Protein expression analysis of the effect of galectin-3 inhibition on CSE-induced EPC autophagy. Galectin-3 protein was examined by Western blotting after EPCs were infected with 3 shRNAs for galectin-3 (shRNA-1, shRNA-2, and shRNA-3) or shRNA-NC (a). EPCs were infected by shRNA-Gal3 (shRNA-1) and shRNA-NC before being treated with 8% of CSE for 24 h, and then Western blots were used to examine p-62, LC3B-I, and LC3B-I (b). 3MA (an autophagy inhibitor) served as a control. Quantification of p-62 (c). Quantification of the LC3B-II/I ratio (d). EPCs were infected by shRNA-Gal3 (shRNA-1) and shRNA-NC, and then Western blots were used to examine p-62, LC3B-I, and LC3B-I (e). Quantification of p-62 (f). Quantification of the LC3B-II/I ratio (g). Data are represented as mean ± SD. *n* ≥ 3. ^∗^*p* < 0.05, ^∗∗^*p* < 0.01, and ^∗∗∗^*p* < 0.001 vs. PBS or the respective group.

**Figure 6:**
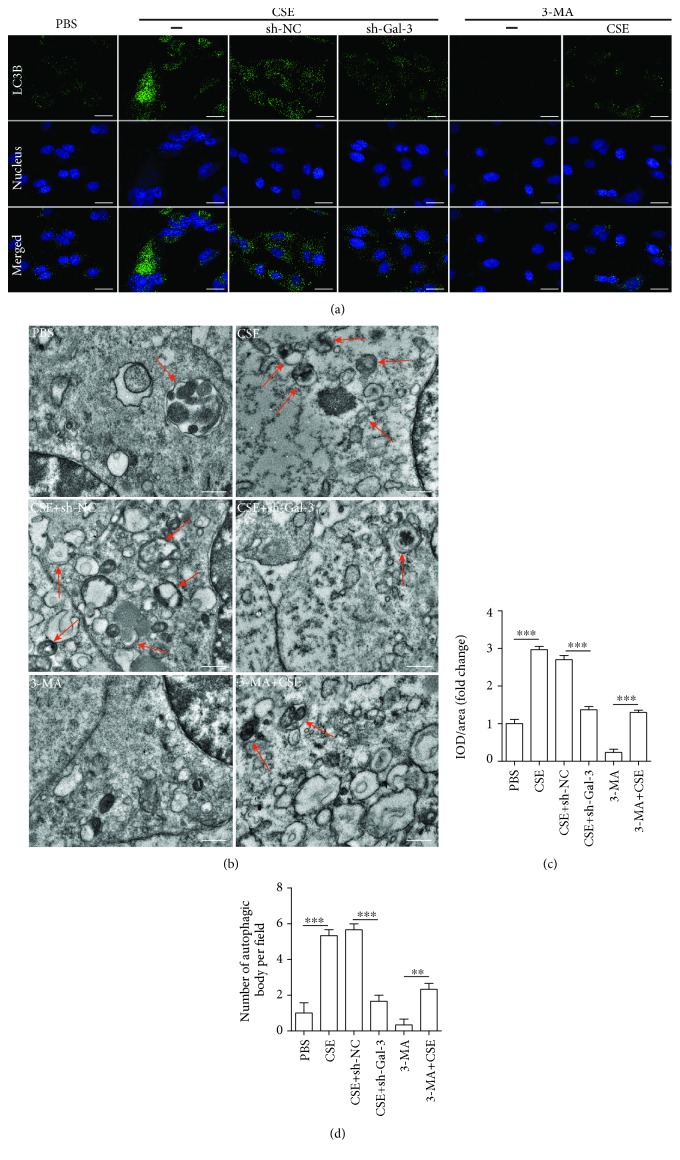
Microscopic observation of the effect of galectin-3 inhibition on CSE-induced EPC autophagy. EPCs were infected by shRNA-Gal3 (shRNA-1) and shRNA-NC before being treated with 8% of CSE for 24 h. 3MA (an autophagy inhibitor) served as a control. Immunofluorescence of LC3B expression on the cell membrane of EPCs in different groups (a) (scale bar = 100 *μ*m). Quantification of IOD/area per field (c). Representative transmission electron microscopy images showing the autophagosomes (red arrows) in EPCs from different groups (b) (scale bar = 1 *μ*m). The number of autophagosomes per cell (d). Data are represented as mean ± SD. *n* ≥ 3. ^∗∗^*p* < 0.00 and ^∗∗∗^*p* < 0.001 vs. the respective group.

In the present study, we investigated whether galectin-3 mediated the effect of CSE on enhancing autophagy of EPCs. EPCs were infected by shRNA-Gal3 or shRNA-NC and then treated with PBS and CSE (8%) for 24 h. First, manipulation of galectin-3 was achieved by transfecting EPCs with sh-RNA-Gal3 to suppress galectin-3 expression, and among 3 sh-RNA-Gal-3 tested, shRNA-1 exerted the greatest effect on downregulating galectin-3 (Figure 5(a)). We thus used shRNA-1 to do the following experiments. We found that downregulation of galectin-3 abrogated the effects of CSE on decreasing p62 protein expression (Figures 5(b) and 5(c)), increasing the IL3B-II/I ratio (Figures 5(b), 5(d), 6(a), and 6(c)), and inducing autophagosome accumulation (Figures 6(b) and 6(d)). Furthermore, we used 3-methyladenine (3-MA) (an autophagy inhibitor) as a positive control and we found that galectin-3 inhibition and 3-MA exerted the similar effects (Figures 5(b)–5(d) and 6(a)–6(d)). In addition, we showed that shRNA-gal-3 alone increased p-62 expression and decreased the LC3B-II/I ratio (Figures 5(e)–5(g)).

### 3.7. Inhibition of Galectin-3 Attenuated CSE-Induced EPC Dysfunction

**Figure 7:**
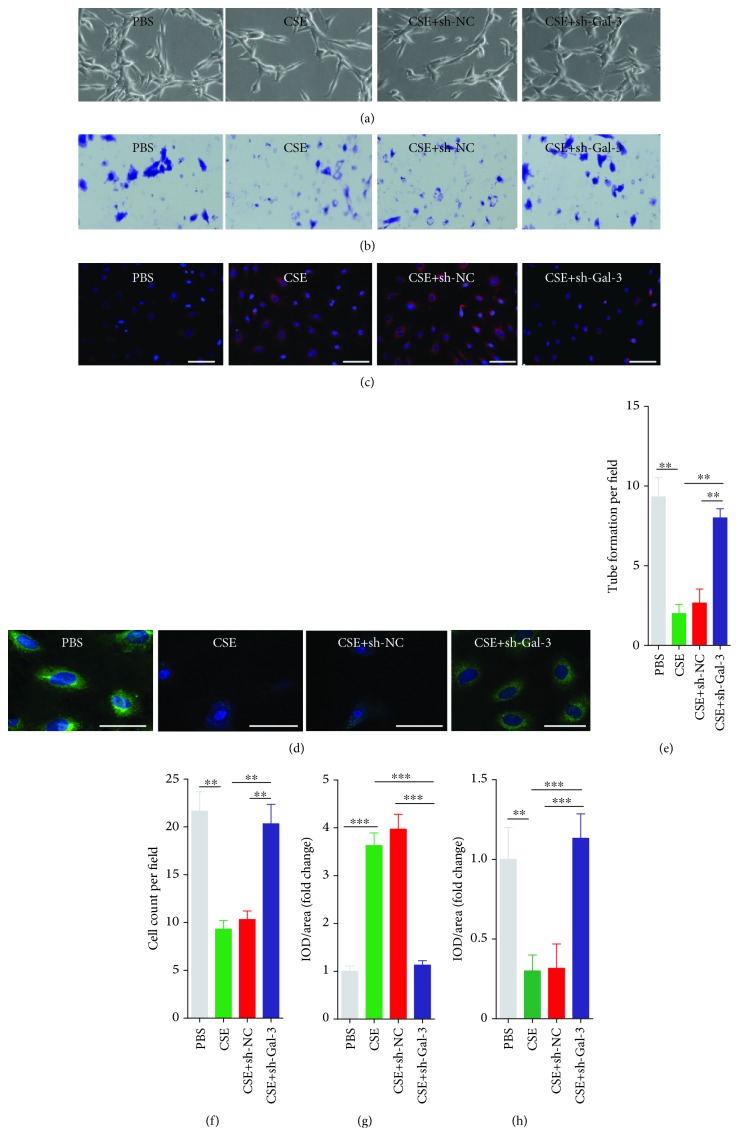
Inhibition of galectin-3 attenuated CSE-induced EPC dysfunction. EPCs were infected by shRNA-Gal3 and shRNA-NC and then treated with PBS, CSE (8%) for 24 h. EPC tube formation capacity was analyzed with a tube formation assay (a). EPC migration capacity was analyzed with a migration assay (b). Intracellular ROS was examined by DHE staining (c). eNOS was examined by IF (d). Quantification of tube formation per field (e). Quantification of cell count per field (f). Quantification of IOD/area per field (ROS) (g). Quantification of IOD/area per field (eNOS) (h). Data are represented as mean ± SD. *n* ≥ 3. ^∗∗^*p* < 0.01 and ^∗∗∗^*p* < 0.001 vs. the respective group. Scale bar = 100 *μ*m.

We also investigated whether galectin-3 mediated the adverse effect of CSE on the functions of EPCs. We found that downregulation of galectin-3 abrogated the effects of CSE on decreasing EPC tube formation capacity (Figures 7(a) and 7(e)) and migration capacity (Figures 7(b) and 7(f)) and increasing intracellular ROS production (Figures 7(c) and 7(g)) and decreasing intracellular eNOS expression (Figures 7(d) and 7(h)).

### 3.8. Galectin-3 Mediates the Regulation of the AMPK/mTOR Signaling Pathway by CSE

**Figure 8:**
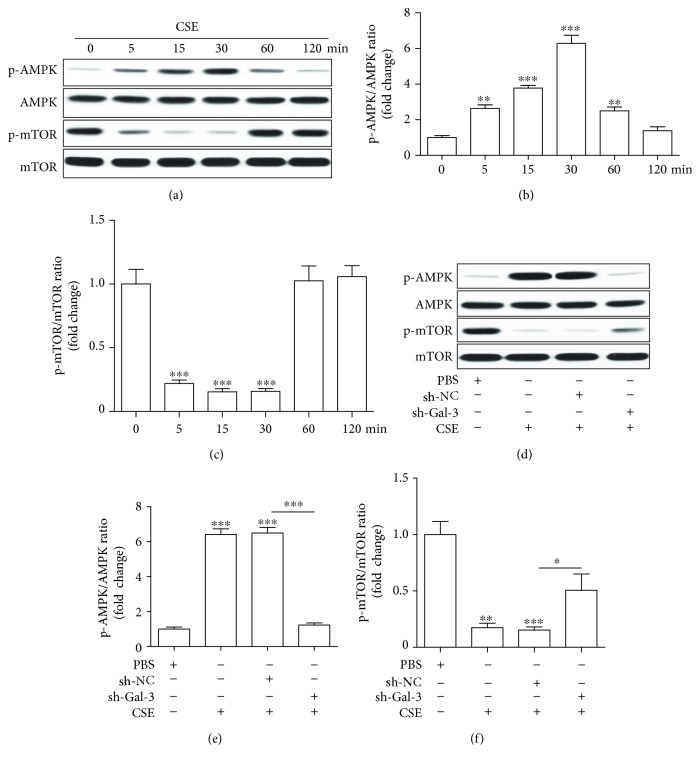
The regulation of AMPK/mTOR by CSE was mediated by galectin-3. EPCs were treated with CSE (8%) for 5, 15, 30, 60, and 120 min (a–c). Western blots of p-AMPK, AMPK, p-mTOR, and mTOR (a) and quantification of p-AMPK (b) and p-mTOR (c). EPCs were infected by shRNA-Gal3 and shRNA-NC and then treated with PBS, CSE (8%) for 30 min (d–f). Western blots of p-AMPK, AMPK, p-mTOR, and mTOR (d) and quantification of p-AMPK (e) and p-mTOR (f). Data are represented as mean ± SD. *n* ≥ 3. ^∗^*p* < 0.05, ^∗∗^*p* < 0.01, and ^∗∗∗^*p* < 0.001 vs. 0 min or PBS or the respective group.

We later examined the involvement of AMPK/mTOR in the effects of CSE on EPCs. First, EPCs were treated with PBS and CSE (8%), and then AMPK/mTOR signaling pathway proteins (p-AMPK, AMPK, p-mTOR and mTOR) were examined by Western blotting at 0, 5, 15, 30, 60, and 120 min (Figure 8(a)). We found that p-AMPK expression was upregulated since 5 min after treatment with CSE and peaked at 30 min (Figure 8(b)), while p-mTOR expression was downregulated since 5 min after treatment with CSE and the greatest downregulation was also seen at 30 min (Figure 8(c)). Then we transfected EPCs with shRNA-1 and sh-NC to examine whether galectin-3 inhibition mediated the regulation of AMPK/mTOR by CSE. We found that downregulation of galectin-3 intervened in the regulation of p-AMPK and p-mTOR expression by CSE (Figures 8(d)–8(f)).

## 4. Discussion

In the present study, we have made several important findings about the effects of CSE on EPC autophagy and dysfunction and the underlying mechanisms. First, we demonstrated that CSE enhanced autophagy and induced dysfunction of EPCs. Second, CSE upregulated galectin-3 expression on EPCs and a shRNAi-mediated knockdown of galectin-3 abrogated CSE-induced EPC autophagy and dysfunction. Third, CSE activated AMPK and inactivated mTOR on EPCs and inhibition of galectin-3 abolished these effects. Taken together, our results suggest that galectin-3 inhibition intervenes in CSE-induced autophagy and dysfunction of EPCs and these effects may involve the AMPK/mTOR signaling pathway. Our results provide a new insight into the mechanism underlying dysfunction of EPCs induced by CSE.

Previous studies showed that CSE (1%, for 24 h) significantly decreased proliferation, adhesion, and secretion capacities of EPCs and decreased eNOS expression [35]. Long-term exposure to nicotine (3 and 6 months) reduced the EPC number and proliferation and migration capacity, in addition to inducing EPC senescence in rats [36]. In our study, we confirmed that 8% CSE for 24 h, a concentration and duration that does not affect cell viability, decreased tube formation and migration capacity and increased intracellular ROS and decreased eNOS expression of EPCs. eNOS can be used as a reflection of endothelium function, since it is the key enzyme to synthesize endothelium-derived nitric oxide which plays a central role in maintaining endothelium homeostasis [37]. ROS directly inactivates endothelium-derived nitric oxide and promotes endothelial dysfunction [37]. So, the effect of CSE on eNOS/ROS is related to its adverse effect of EPC function.

Cigarette smoking-induced autophagic disruption has been reported in atherosclerosis [38], lung cancer [39], pulmonary fibrosis [40], age-related macular degeneration [24], and chronic obstructive pulmonary disease [41]. Csordas et al. reported that CSE induced autophagic cell death in human umbilical vein endothelial cells [42]. We found that CSE enhanced autophagy in EPCs, which was characterized by a decrease in p62 protein, an increase in the LC3B-II/I ratio, and accumulation of autophagosomes. Our results suggest that excessive autophagy induced by CSE may be linked to EPC dysfunction, preceding cell death of EPCs.

Galectin-3 plays an important role in multiple cellular physiological and pathological processes including cell growth, apoptosis, angiogenesis, and inflammation [26, 27]. Recent studies suggested the role of galectin-3 in autophagic response [32, 33]. Galectin-3 mediated autophagy to protect cells against endomembrane damage associated with lysosomal dysfunction [32], and endogenous galectin-3 protected intracellular listeria monocytogenes by suppressing the autophagic response in mouse macrophages infected with listeria monocytogenes [33]. So, in the present study, we studied the involvement of galectin-3 in the effects of CSE on autophagy and dysfunction of EPCs. We first found that CSE upregulated galectin-3 gene and protein expression on EPCs. A previous study also reported that CSE induced the release of galectin-3 in airway epithelial cells, which was higher in chronic obstructive pulmonary disease-derived cells compared with control-derived cells [43]. We further found that knockdown of galectin-3 abrogated CSE-induced autophagy and dysfunction of EPCs and these effects were similar to the effects exerted by 3-MA. So, upregulation of galectin-3 plays an important role in mediating autophagy and dysfunction of EPCs induced by cigarette smoking.

The AMPK/mTOR signaling pathway is an important regulator of cell cycle [44], mitochondrial function [45], and metabolism activities [46, 47]. The AMPK/mTOR signaling pathway also tightly regulates autophagy as shown in many studies. Metastasis of renal cancer cells was prevented by inducing autophagy via the AMPK/mTOR signaling pathway through using thymoquinone [48]. Bone resorption was inhibited by enhancing autophagy via the AMPK/mTOR signaling pathway under the condition of using osteoprotegerin [49]. Myocardial ischemia/reperfusion injury was attenuated by inhibiting autophagy via the AMPK/mTOR signaling pathway via adding metformin [50]. The AMPK/mTOR signaling pathway was also demonstrated to play a big role in smoking-induced autophagic cell death [47]. In the present study, we found that CSE activated AMPK and inactivated mTOR, suggesting that AMPK/mTOR is likely involved in CSE-induced autophagy and dysfunction of EPCs. We also found that inhibition of galectin-3 abolished CSE's effects on activating AMPK and inhibiting mTOR, indicating that galectin-3 mediates CSE's effect on the AMPK/mTOR signaling pathway. This is supported by a very recent paper showing that galectins control autophagy via AMPK/mTOR in response to lysosomal damage, a strong inducer of autophagy [51]. Taken together, galectin-3 is an important mediator for ESC-induced autophagy and dysfunction of EPCs via controlling the AMPK/mTOR signaling pathway. Future studies are needed to examine the beneficial effect of blocking galectin-3 on smoke-induced EPC dysfunction in animals or patients.

In conclusion, our results suggest that galectin-3 inhibition alleviates CSE-induced autophagy and dysfunction of EPCs and these effects may be regulated by the AMPK/mTOR signaling pathway. Blockade of galectin-3 may be a potential therapeutic target for inhibiting CSE-induced EPC dysfunction and dysregulated autophagy.

## Figures and Tables

**Table 1:** PCR primer used for cDNA.

Genes	Primers
Galectin-3	F: 5′-TATAAGATCTGAGGATAGGTGGGTTCCCGAGAACT-3′
	R: 5′-ATATGAATTCTCTCAGGGCTATGCCGCCTAAGTAC-3′
GAPDH	F: 5′-GACAGTCAGCCGCATCTTCT-3′
	R: 5′-TTAAAAGCAGCCCTGGTGAG-3′

## Data Availability

The data used to support the findings of this study are available from the corresponding author upon request.
